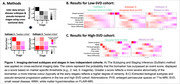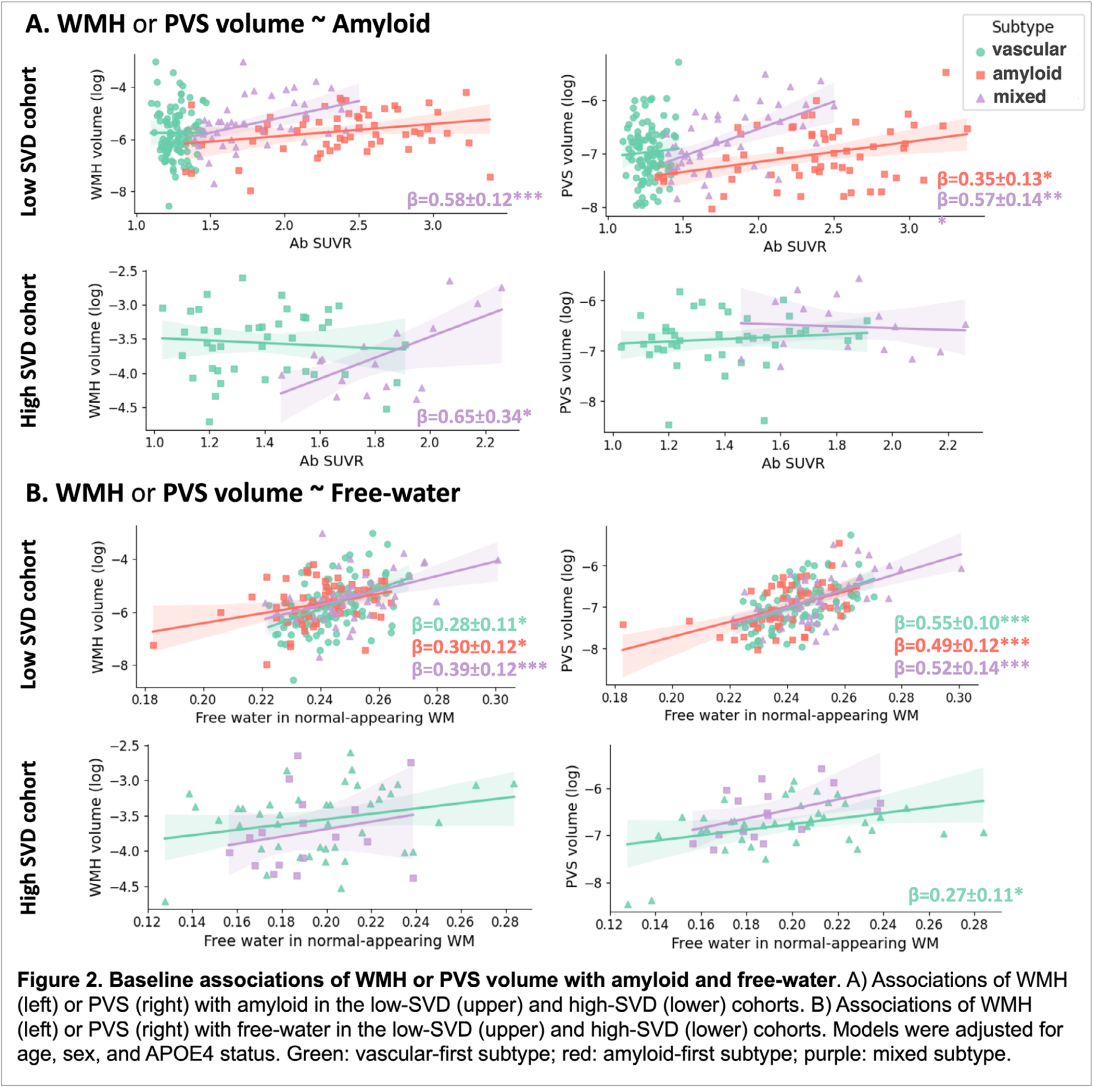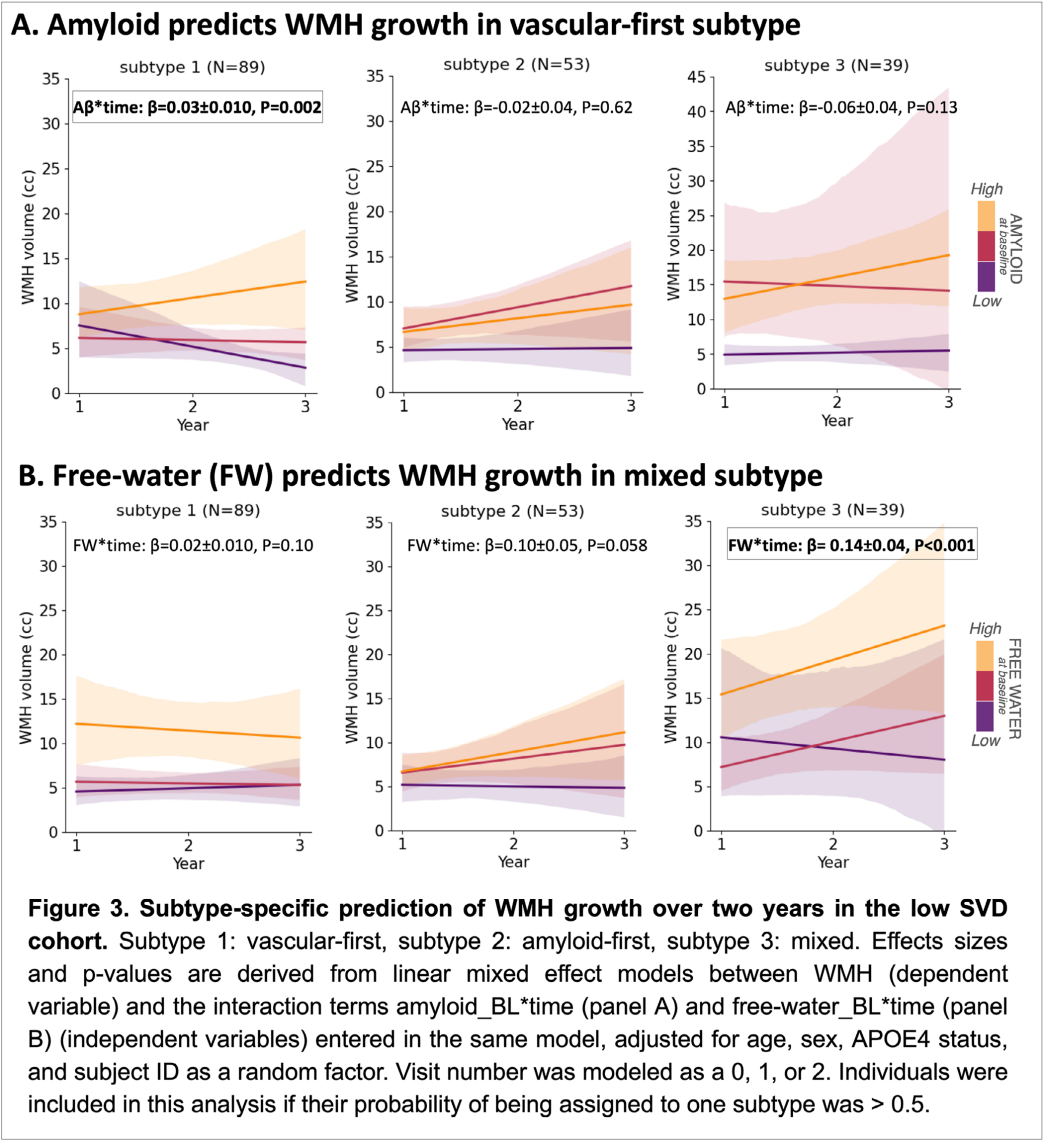# Heterogeneity and progression of amyloid and vascular injury in Alzheimer's and Mixed dementia cohorts

**DOI:** 10.1002/alz70856_100574

**Published:** 2025-12-25

**Authors:** Julie Ottoy, Andrew Clappison, Eric Yin, Min Su Kang, Erin Gibson, Katrina Carver, Joel Ramirez, Miracle Ozzoude, Katherine Zukotynski, Stephanie Berberian, Ginelle J. Feliciano, Christopher JM Scott, Walter Swardfager, Fuqiang Gao, Lauren Abby Woods, Eric E. Smith, Nesrine Rahmouni, Ging‐Yuek Robin Hsiung, Robert Laforce, Frank S. Prato, Phillip H. Kuo, Jean‐Paul Soucy, Jean‐Claude Tardif, Pedro Rosa‐Neto, Sandra E. Black, Maged Goubran

**Affiliations:** ^1^ Dr. Sandra E. Black Centre for Brain Resilience and Recovery, Toronto, ON, Canada; ^2^ LC Campbell Cognitive Neurology Research Unit, Sunnybrook Research Institute, University of Toronto, Toronto, ON, Canada; ^3^ Dr. Sandra E. Black Centre for Brain Resilience and Recovery, LC Campbell Cognitive Neurology, Hurvitz Brain Sciences Program, Sunnybrook Research Institute, University of Toronto, Toronto, ON, Canada; ^4^ Schulich School of Medicine and Dentistry, Western University, Toronto, ON, Canada; ^5^ Hotchkiss Brain Institute, University of Calgary, Calgary, AB, Canada; ^6^ Translational Neuroimaging Laboratory, The McGill University Research Centre for Studies in Aging, Montréal, QC, Canada; ^7^ Djavad Mowafaghian Centre of Brain Health, Vancouver, BC, Canada; ^8^ Clinique Interdisciplinaire de mémoire, CHU de Québec ‐ Université Laval, Quebec City, QC, Canada; ^9^ Schulich School of Medicine and Dentistry, Western University, London, ON, Canada; ^10^ Department of Medical Imaging, Medicine, and Biomedical Engineering, University of Arizona, Tucson, AZ, USA; ^11^ Montreal Neurological Institute, McGill University, Montréal, QC, Canada; ^12^ Montreal Heart Institute, McGill University, Montreal, QC, Canada; ^13^ McConnell Brain Imaging Centre, Montreal Neurological Institute, McGill University, Montreal, QC, Canada

## Abstract

**Background:**

Alzheimer's disease (AD) is a heterogeneous disorder that is often comorbid with cerebral small vessel disease (SVD). Previous studies used highly characterized AD cohorts to identify imaging‐derived subtypes to explain patient heterogeneity. Here, we studied two distinct dementia cohorts (one with a low SVD burden and one heterogeneous cohort with a Fazekas score >2) to define imaging‐derived subtypes. We then determined, within each subtype, if amyloid or free‐water could predict vascular lesions at baseline and over time.

**Method:**

We studied 262 individuals across two cohorts. The longitudinal TRIAD (“low‐SVD”) cohort included cognitively normal, MCI, and AD dementia (baseline, year2, year3: *N* = 202, 100, 70). The MITNEC‐C6 (“high‐SVD”) cohort included real‐world patients with mixed dementia and moderate‐to‐severe periventricular white matter hyperintensity (WMH) burden (*N* = 60). We quantified WMH and enlarged perivascular space (PVS) volumes based on FLAIR‐MRI and T1w‐MRI, respectively, in the white matter using our novel deep learning segmentation tool segCSVD (Gibson et al. 2024 HBM). Disease subtypes were identified through the Subtype and Stage Inference (SuStaIn) algorithm (Figure 1A) using the following markers: 18F‐AZD4694 or 18F‐AV45 amyloid‐SUVR in the AD‐signature regions, total WMH, total PVS, basal ganglia PVS, and DTI‐derived free‐water, fractional anisotropy and mean diffusivity in the normal‐appearing white matter.

**Result:**

Both cohorts showed a ‘vascular‐first’ subtype (green) and a ‘mixed’ subtype (purple) (Figure 1B‐C). The low‐SVD cohort additionally showed an ‘amyloid‐first’ subtype (red). In both cohorts, greater amyloid was significantly associated with greater WMH volume in the ‘mixed’ subtypes (Figure 2A). Both cohorts also showed a positive association of free‐water with WMH and PVS volume in each of the subtypes (Figure 2B). Finally, greater baseline amyloid predicted faster WMH growth in the ‘vascular‐first’ subtype (Figure 3A). Whereas, in the ‘mixed’ subtype, greater baseline free‐water but not amyloid predicted faster WMH growth (Figure 3B). Neither amyloid nor free‐water predicted WMH growth in the ‘amyloid‐first’ subtype nor did they predict PVS growth.

**Conclusion:**

In the mixed subtype, which is likely the most common subtype in memory clinics and community‐based samples, amyloid was associated with WMH volume at baseline, but greater free‐water levels predicted WMH growth over time.